# 11p15 DNA-methylation analysis in monozygotic twins with discordant intrauterine development due to severe twin-to-twin transfusion syndrome

**DOI:** 10.1186/1868-7083-6-6

**Published:** 2014-03-28

**Authors:** Felix Schreiner, Bettina Gohlke, Sonja Stutte, Peter Bartmann, Kurt Hecher, Johannes Oldenburg, Osman El-Maarri, Joachim Woelfle

**Affiliations:** 1 Pediatric Endocrinology Division, Children’s Hospital, University of Bonn, Adenauerallee 119, 53113 Bonn, Germany; 2 Department of Neonatology, Children’s Hospital, University of Bonn, Adenauerallee 119, 53113 Bonn, Germany; 3 Department of Obstetrics and Fetal Medicine, University Medical Center Hamburg-Eppendorf, Martinistraße 52, 20246 Hamburg, Germany; 4 Institute for Experimental Hematology and Transfusion Medicine, University of Bonn, Sigmund-Freud-Straße 25, 53127 Bonn, Germany

## Abstract

**Background:**

Prenatal growth restriction and low birth weight have been linked to long-term alterations of health, presumably via adaptive modifications of the epigenome. Recent studies indicate a plasticity of the 11p15 epigenotype in response to environmental changes during early stages of human development.

**Study design:**

We analyzed methylation levels at different 11p15 loci in 20 growth-discordant monozygotic twin pairs. Intrauterine development was discordant due to severe twin-to-twin transfusion syndrome (TTTS), which was treated by fetoscopic laser coagulation of communicating vessels before 25 weeks of gestation. Methylation levels at age 4 were determined in blood and buccal cell-derived DNA by the single nucleotide primer extension reaction ion pair reverse-phase high performance liquid chromatography (SNuPE IP RP HPLC) assay. Methylation at LINE-1 repeats was analyzed as an estimate of global methylation.

**Results:**

In general, variance of locus-specific methylation levels appeared to be higher in buccal cell- as compared to blood cell-derived DNA samples. Paired analyses within the twin pairs revealed significant differences at only one CpG site (IGF2 dmr0 SN3 (*blood*), +1.9% in donors; *P* = 0.013). When plotting the twin pair-discordance in birth weight against the degree of discordance in site-specific methylation at age 4, only a few CpGs were found to interact (one CpG site each at IGF2dmr0 in blood/saliva DNA, one CpG at LINE-1 repeats in saliva DNA), with 26 to 36% of the intra-twin pair divergence at these sites explained by prenatal growth discordance. However, across the entire cohort of 40 children, site-specific methylation did not correlate with SD-scores for weight or length at birth. Insulin-like growth factor-II serum concentrations showed significant within-twin pair correlations at birth (R = 0.57) and at age 4 (R = 0.79), but did not differ between donors and recipients. They also did not correlate with the analyzed 11p15 methylation parameters.

**Conclusion:**

In a cohort of 20 growth-discordant monozygotic twin pairs, severe alteration in placental blood supply due to TTTS appears to leave only weak, if any, epigenetic marks at the analyzed CpG sites at 11p15.

## Background

The association between low birth weight and an increased risk of developing metabolic and cardiovascular disease later in life has been known for decades [1]. However, the molecular mechanisms underlying the phenomenon of fetal programming remained largely unknown. In recent years, an increasing number of studies identified epigenetic alterations at certain loci to be involved in this process of programming and adaptation [2-5].

The 11p15 chromosome region harbors a set of imprinted genes involved in the expression of insulin-like growth factor (IGF)-II and fetal growth. Gene expression at this locus is controlled by differentially methylated regions (dmr*s*), and disturbances of these control elements resulting from either genetic or epigenetic mutations are known to cause fetal growth disorders such as Beckwith-Wiedemann syndrome (BWS) or Silver-Russell syndrome (SRS) [6]. Tissue-specific 11p15 imprinting abnormalities have also been implicated in the development of different human tumors [7,8]. Interestingly, Heijmans and colleagues [9] reported on persistent epigenetic differences at the 11p15 locus among adults six decades after periconceptional exposure to nutrient restriction during the Dutch famine in the winter of 1944 to 1945, and subsequent studies revealed folic acid supply before conception and during pregnancy to be associated with the methylation pattern at the 11p15 region in infants [10,11].

Here, we analyzed the methylation status at different 11p15 regions in a cohort of monozygotic twin pairs discordant for prenatal growth due to a severe twin-to-twin transfusion syndrome (TTTS). TTTS twins suffer from a substantial asymmetry in fetal blood supply caused by communicating placental vessels, which can lead to hypervolemia, heart insufficiency and hydrops fetalis in the recipient, and to critical hypovolemia, nutrient restriction and growth arrest in the donor twin. Since the 1990s, endoscopic laser coagulation of the communicating vessels has become a standard treatment option in many industrialized countries worldwide [12,13]. Although still a medical challenge, TTTS twins offer a unique goal to analyze the influence of prenatal environmental changes on the epigenome.

## Methods

### Twin cohort

We analyzed 20 monozygotic twin pairs with discordant intrauterine growth due to severe TTTS. In brief, TTTS results from communicating placental vessels and threatens the donor’s and recipient’s health by either hypovolemia, anhydramnios, nutrient restriction and growth retardation, or hypervolemia, heart insufficiency and hydrops fetalis. Fetoscopic laser coagulation of the communicating placental vessels was performed before 25 weeks of gestation in all 20 pregnancies (range 17.1 to 24.9 weeks). Further information on treatment regime and study design is given elsewhere [13-15]. Mean age at birth was 34.8 weeks of gestation (SD ± 2.1 weeks; range 29.7 to 37.4 weeks). Mean birth weight was 1,970 g (SD ± 500 g; range 790 to 3,060 g). Birth weight differences between donor and recipient ranged from 0 to 62% (mean 20.5%). On examination, mean age of the children was 4.4 years (SD ± 0.6 years; range 2.7 to 5.1 years). Auxological data including calculations of intra-twinpair differences were expressed as standard deviation score (SDS) according to national reference percentiles ([16,17]; Table 1). At birth, parameters between donor and recipient were classified as discordant if either birth weight difference was ≥10% [18] or birth length differed by ≥1.0 (SDS). At age 4, classification of discordance was based on body length (SDS) only.

**Table 1:** Auxological parameters at birth and at age 4 years according to the former twin-to-twin transfusion syndrome status

	**Recipient**	**Donor**	***P***
Gestational age at laser treatment (weeks)	20.96 ± 2.27		
Gestational age at birth (weeks)	34.54 ± 2.16		
Birth weight (g)	2,141 ± 428	1,780 ± 522	<0.001
Birth weight SDS	-0.62 ± 0.80	-1.51 ± 0.91	<0.001
Birth length (cm)	45.42 ± 3.08	42.63 ± 4.28	<0.001
Birth length SDS	-0.48 ± 1.11	-1.47 ± 1.27	<0.001
Age at follow-up	4.41 ± 0.59		
Height SDS 4 years	-0.39 ± 0.86	-1.01 ± 1.10	<0.001
Weight SDS 4 years	-0.14 ± 0.67	-0.97 ± 0.83	<0.001
BMI SDS 4 years	-0.11 ± 0.81	-0.74 ± 0.71	0.001
IGF-II in cord blood (ng/ml)	322.69 ± 57.92	322.81 ± 44.97	0.995
IGF-II at age 4 years (ng/ml)	533.10 ± 95.82	539.95 ± 98.69	0.825

Data are given as mean ± SD. BMI, body mass index; IGF, insulin-like growth factor; SDS, standard deviation score.

Written informed consent was obtained from the twins’ parents. The study was approved by the ethics committee of the University of Bonn.

### Hormone measurements

IGF-II serum levels in serum samples were determined by a commercially available RIA kit (Mediagnost, Germany). Neonatal hormone measurements from 16 out of 20 twin pairs of the current study cohort have been included in previous reports focusing on the impact of impaired prenatal growth on the physiology of IGF-I and -II [14,19].

### Quantitative methylation analysis

DNA from blood and saliva samples was extracted using commercially available kit protocols (QiaAmp DNA Blood®, Qiagen, Hilden, Germany; Oragene®, DNA Genotek, Ottawa, Canada). Whereas blood-derived DNA was available from all 20 twin pairs, suitable amounts of saliva DNA were obtained in only 34 of 40 childen (16 complete twin pairs). For methylation analysis, a total of 1 μg DNA was chemically modified by bisulfite conversion using the Epitect® kit (Qiagen). The basic principle of bisulfite modification is the chemical conversion of unmethylated cytosine residues to uracil, whereas methylated cytosines remain unchanged [20]. This step allows accurate quantitative measurement of locus-specific cytosine methylation by several PCR-based downstream reactions [21-23].

Locus-specific methylation was determined at several CG dinucleotides within the H19 and IGF2 differentially methylated regions and the KCNQ1OT1 promoter using the SIRPH (SNuPE IP RP HPLC) assay. A detailed description of this method is given elsewhere [23]. In brief, a single nucleotide primer extension reaction (SNuPE) of bisulfite-converted DNA followed by ion pair reverse-phase high performance liquid chromatography (IP RP HPLC) enables discrimination and quantitative assessment of formerly methylated versus unmethylated CpGs depending on specific mass and hydrophobicity of the extended primer product.

**Figure 1:**
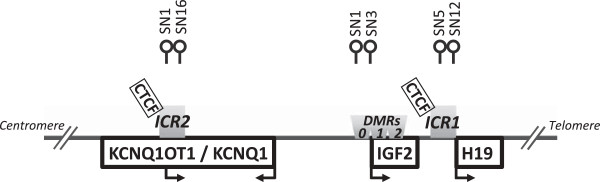
**Location of the analyzed CpGs at the 11p15.5 region.** Exact positions of the target CpGs as well as nucleotide sequences of the amplification and extension primers used in the single nucleotide primer extension reaction ion pair reverse-phase high performance liquid chromatography (SnuPE IP RP HPLC) assay are listed in Additional file 1: Table S1. DMR, differentially methylated region; ICR, imprinting control region.

Figure 1 displays the positions of the analyzed CpG sites at the chromosome region 11p15.5. Exact target CpG site positions and nucleotide sequences of amplification and extension primers used in the SNuPE IP RP HPLC assay are listed in Additional file 1: Table S1. Selection of target CpG sites was based on methodological (avoidance of further CpG dinucleotides within the extension primer complementary regions) and functional aspects. SN is the internal abbreviation for the SNuPE extension primers used; the SN-number corresponds to the relative position of the CpG site within the PCR amplicon. CpG sites SN1 and SN3 at the IGF2 dmr0 region are identical with CpG sites 1 and 3 in the study of Hoyo and colleagues [11] and have also been analyzed by Hejmans and co-workers in their Dutch famine cohort [9]. CpG sites targeted with H19 SN5 and SN12 are located within the H19 promoter region and a CTCF6 binding site approximately 800 bp upstream of the transcription start site. The CpG sites at KCNQ1OT1 (SN16 and SN1) are located in a CpG island surrounding the transcription start site of the antisense KCNQ1OT1 transcript. This CpG Island shows a relatively uniform pattern of methylated maternal and unmethylated paternal alleles, with loss of maternal methylation in many patients with Beckwith-Wiedemann syndrome [24]. Because of its high CpG-density and difficulties with the selection of CpG-free amplification and extension primers, methylation levels at this region were analyzed using the corresponding 3′5′ bisulfite DNA strand, explaining the reversed order of appearance (SN16, SN1) in text and figures. Extension primers SN1 and SN13 for the assessment of LINE-1 methylation are identical to extension primers SN9 and SN8 used in a previous study [25]. The term “mean methylation” at a specific region refers to the average methylation levels calculated from ((SNA + SNB)/2).

### Statistical analysis

Data analyses were performed using the SPSS software version 20 (SPSS IBM, Armonk, NY, USA). Unless otherwise defined, auxological and biochemical data, including intra-twin pair differences are expressed as mean ± SD. Differences between groups and between twin pairs were analyzed by analysis of variance (ANOVA), Student’s *t* test and Mann-Whitney U-test. Relations within twin pairs were examined by paired *t* tests and correlation analyses (Spearman; Pearson). *P* values < 0.05 were considered statistically significant.

## Results

### Auxological parameters and circulating insulin-like growth factor-II levels

Detailed information on auxological development and hormone measurements in serum samples drawn at birth and at the follow-up examination 4 years later is given elsewhere [14,15]. In brief, 11/20 pairs had differences in birth weight of ≥10% or in birth length of ≥1.0 SDS. At a mean age of 4.4 years, only 5/20 pairs were still discordant for body length. Auxological parameters of the current cohort are displayed in Table 1. As reported earlier, birth weight differences and *IGF-I* concentrations in cord blood were significantly associated with the growth pattern during the first 4 years of life [19].

In the initial study cohort consisting of 27 twin pairs, IGF-II concentrations in cord blood showed a relatively strong intra-twin pair correlation (R = 0.58; *P* < 0.01) [14]. Although the majority (16/20) of twin pairs of the current cohort have been part of this initial collective, a similar strong correlation (R = 0.57; *P* < 0.05) was detected only after excluding three outlier pairs with the highest discordance for cord blood IGF-II levels (delta 100 ng/ml or higher). IGF-II cord blood concentrations were not different between donors and recipients (Table 1). They did not correlate with SD scores for weight or length at birth, and intra-twin pair differences in cord blood IGF-II levels were also not related to the degree of discordance in birth weight or birth length SDS (all *P* > 0.2).

At age 4, the IGF-II intertwin correlation was markedly stronger (total cohort R = 0.79; *P* < 0.01; Additional file 2: Figure S1). However, neither IGF-II concentrations nor intertwin differences correlated significantly when comparing neonatal values against those determined at age 4 years. There were also no differences between the donors’ and recipients’ IGF-II concentrations at age 4 (Table 1). Neither IGF-II concentrations at birth nor those determined at the follow-up examination correlated significantly with any of the following variables: gestational age at laser treatment, gestational age at birth, birth weight or birth length (all *P* > 0.2).

### Methylation analyses

#### Variability of methylation levels across different 11p15 regions and tissues

**Figure 2:**
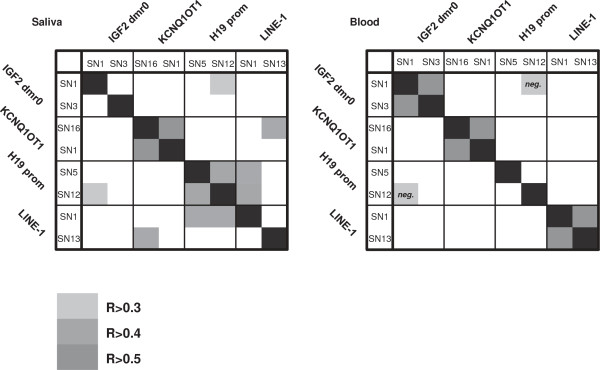
**Intra-individual correlation of single CpG methylation levels within and between regions.** Spearman’s correlation coefficients are indicated graphically. Correlation coefficients within regions were: saliva - KCNQ1OT1, R = 0.814, *P* < 0.01; H19, R = 0.527, *P* < 0.01; blood - IGF2 dmr0, R = 0.559, *P* < 0.01; KCNQ1OT1, R = 0.748, *P* < 0.01; LINE-1, R = 0.539, *P* < 0.01). Significant correlations or trends (*P* < 0.1) for relations within one sample but between regions were: saliva - IGF2 dmr0 SN1 × H19prom SN12, R = 0.335, *P* = 0.075; KCNQ1OT1 - SN16 × LINE-1 SN1, R = 0.410, *P* = 0.016; H19prom SN5 × LINE-1 SN13, R = 0.400, *P* = 0.031; H19prom SN12 × LINE-1 SN13, R = 0.469, *P* = 0.010; blood - IGF2 dmr0 SN1 × H19prom SN12, R = -0.329, *P* = 0.038).

For each analyzed 11p15 region, methylation levels of two separate CpG sites were determined by the quantitative SNuPE IP RP HPLC assay. When comparing methylation levels between two CpG dinucleotides within one sample and one region, we detected significant correlations for most regions in either saliva or blood DNA (Figure 2). However, only a few CpG sites showed significant interactions across different 11p15 regions (Figure 2) and, with the exception of one LINE-1-CpG (LINE-1 SN13 blood versus saliva R = 0.468, *P* < 0.01), we also did not observe significant intra-individual correlations across different tissues (data not shown). In general, variance of methylation levels appeared to be markedly higher in saliva as compared to blood DNA. This is also reflected by generally higher intra-twin pair differences at the majority of CpG sites in saliva-derived DNA, regardless of the status of discordance for auxological parameters at birth (Additional file 1: Table S2). Accordingly, inter-twin correlations were stronger in blood- as compared to saliva-derived DNA samples (Additional file 1: Table S3).

#### Methylation levels according to timing of laser treatment, age and gender

Gestational age at laser treatment and at birth did not correlate significantly with methylation levels or the degree of intra-twin pair methylation differences at any of the analyzed CpGs. In our cohort with a comparatively small age range (2.7 to 5.1 years) we also did not observe significant relations between age at follow-up and methylation levels or the degree of intra-twin pair methylation differences.

As previously reported in adult cohorts [26], LINE-1 methylation levels at CpG site SN13 were slightly higher in male compared to female individuals (blood - SN13, 57.80 ± 0.80% versus 57.16 ± 0.56%, *P* < 0.01; SN1 + SN13/2, 53.86 ± 0.69% versus 53.44 ± 0.49% *P* < 0.05; SN1, not different; saliva - SN13, 61.12 ± 1.38% versus 59.82 ± 0.82%, *P* < 0.01; SN1 + SN13/2, 55.81 ± 0.73% versus 55.12 ± 0.60%, *P* < 0.01; SN1, not different). A significant gender effect was also found for one of two CpG sites at the IGF2 dmr0 (blood - SN3, 39.09 ± 3.24% in boys versus 41.32 ± 3.28% in girls, *P* < 0.05; SN1, not significant; saliva - SN1 and SN3, not different).

#### Methylation levels according to the TTTS (twin-to-twin transfusion syndrome) status (donor versus recipient)

**Figure 3:**
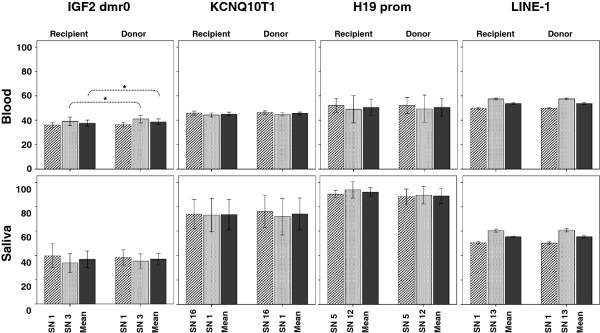
**Site-specific methylation levels (mean ± SD) in blood- and saliva-derived DNA.** Except for IGF2 dmr0 SN3 methylation (*P* = 0.013, paired *t* test) and IGF2 dmr0 average (= SN1 + SN3/2) methylation (*P* = 0.027, paired *t* test) there were no significant differences between former recipients and donors.

The primary aim of our study was to compare locus-specific methylation levels between genetically identical twins with special consideration of their discordant growth during early developmental stages. However, mean methylation levels were largely comparable between recipients and donors (Figure 3). Paired analyses revealed significant differences for only one out of eight analyzed CpG sites (IGF2 dmr0_*blood*_ SN3: 39.16 ± 3.46% in recipients versus 41.03 ± 3.17% in donors, *P* = 0.013, paired *t* test) and only one out of four regions when analyzing average methylation values (IGF2 dmr0_*blood*_ (SN1 + SN3/2): *P* = 0.027, paired *t* test). Subgroup analyses in pairs with either concordance or discordance for auxological parameters at birth (9 versus 11 pairs) or at age 4 (15 versus 5 pairs) did not accentuate these findings (data not shown). Considering the presumed functional interrelation within and between the analyzed 11p15 region, a stringent correction for multiple testing may overestimate the false discovery rate. By setting the number of independent tests to n = 3 regions, the difference observed at IGF dmr0_*blood*_ SN3 would still reach a Bonferroni-adjusted significance level of *P* = 0.017.

**Figure 4:**
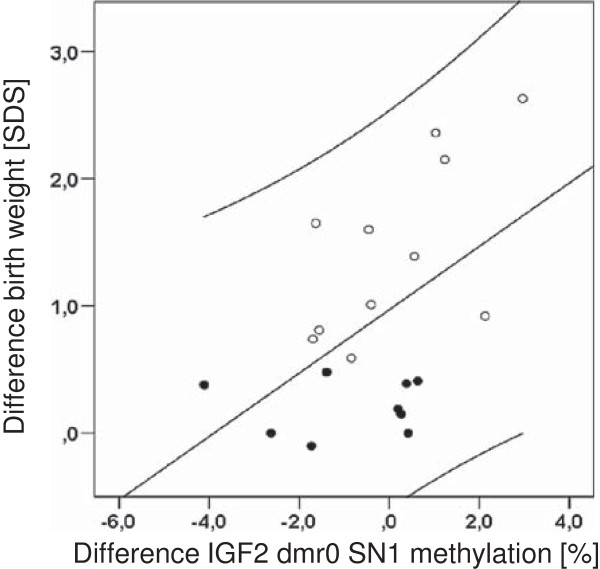
**Relation of inter-twin differences for birth weight and IGF2 dmr0 SN1 methylation; Spearman’s ρ = 0.51 (*P*** **< 0.05).** Filled circles, concordant pairs; open circles, discordant pairs. Note that due to the definition of discordance (difference in birth weight ≥10% and/or birth length ≥1.0 SDS) some pairs with birth weight differences <1.0 SDS were classified as discordant. SDS, standard deviation score.

**Figure 5:**
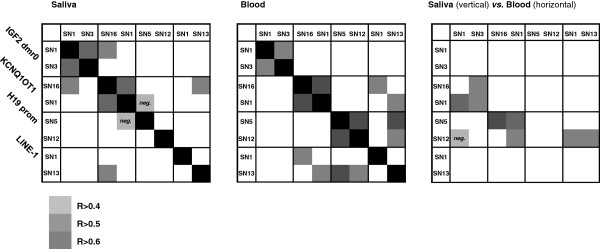
**Pearson correlation coefficients of intra-twin pair methylation differences within and between regions.** Note that the majority of correlation coefficients have a positive value, indicating that intra-twin pair methylation differences within and between regions arise with consistent directionality (that is, increasing difference (= methylation recipient minus methylation donor) at one CpG going along with increasing difference at another CpG).

When plotting the degree of discordance in SD scores for birth weight or length against differences in methylation levels at age 4 years, again only a few CpGs were found to interact: intra-twin pair variation at IGF2 dmr0_*blood*_ SN1, IGF2 dmr0_*saliva*_ SN3, and LINE-1_saliva_ SN13 (up to one outlier pair excluded) revealed significant correlations with discordance in weight and/or length at birth (R-values between 0.51 and 0.60, *P* < 0.05), such that 26 to 36% of the within twin-pair variance in methylation at these sites may be explained by prenatal growth discordance in this simplified view (exemplified in Figure 4). However, according to the above-mentioned definition, discordance for body length and/or weight at birth was present in only 11 out of 20 twin pairs, and the individual extent of catch-up growth between laser treatment and birth may not necessarily reflect the severity and discordance in placental blood flow before treatment. Assuming that variation in locus-specific methylation patterns in response to environmental changes occurs with a consistent directionality in neighboring CpG sites and/or interacting regions, we correlated intra-twin pair methylation differences within and between regions. Indeed, the majority of Pearson correlation coefficients showed positive values, indicating that methylation differences within and between regions in our twin cohort arose with a consistent directionality (Figure 5).

#### Methylation levels and IGF-II serum concentrations

Finally, we compared IGF-II concentrations in cord blood and in samples taken at age 4 years with site-specific 11p15 methylation levels, but did not detect significant correlations (Spearman correlations; all *P* > 0.2; up to two outliers excluded). Similarly, intra-twin pair differences in IGF-II levels did not correlate with intra-twin pair methylation differences (*P* > 0.2).

## Discussion

Studies of twins have driven the exploration of genetics and heritability for a long time and continue to do so hand-in-hand with recent technological advances in the field of developmental programming and epigenetics. Monozygotic twins with a discordant clinical phenotype provide a unique opportunity to evaluate the contribution of environmental factors against the identical genetic background [27-31]. In this study, we have analyzed locus-specific CpG methylation at the 11p15 region in monozygotic twins with severely discordant prenatal development due to TTTS. However, we found only weak evidence for a contribution of environmental factors such as inequality of mid-gestational blood supply to the 11p15 epigenotype at age 4. Pairwise comparisons between former donors and recipients revealed only slight methylation differences at one out of three analyzed 11p15 regions (IGF2 dmr0). Accordingly, correlating the degree of birth weight discordance against variation in locus-specific methylation within twin pairs revealed a significant interaction only for IGF2 dmr0. Overall, we did not observe a significant relation between size at birth and the 11p15 methylation pattern. We conclude that severe alteration in placental blood supply due to TTTS during mid-gestation appears to leave only weak, if any, locus-specific epigenetic marks at the analyzed 11p15 regions.

Although it is generally assumed that severe 11p15 methylation abnormalities, such as loss of methylation at H19, are both an underlying cause and restricted to patients with SRS or SRS-like phenotypes [32-34], measurable variation of the 11p15 methylation pattern arising in response to environmental changes has been described in cohorts of various ages, including very early developmental periods [9-11,31,35]. Heijmans and co-workers reported on persistent epigenetic marks at this region following periconceptional famine exposure, supporting the idea that sufficient periconceptional folic acid supply is essential to establish the 11p15 epigenotype [9,10]. Maternal folic acid intake during pregnancy has also been linked to the 11p15 methylation status in offspring [11,36]. However, findings of other recent studies on the relationship between maternal folate supplementation and global and/or site-specific methylation are controversial, and it is not known whether the subtle methylation changes found in some of these studies would significantly alter gene transcription [36-39]. In addition, genotype-epigenotype interactions have been reported to account for a significant proportion of the variability of methylation levels at the IGF2 dmr0 [40-43].

Our results, as well as data from other recent studies, do not support the idea that intrauterine growth retardation and/or being born small for gestational age without features of SRS are associated with substantial epigenetic changes at the 11p15 locus. Tobi and colleagues [44] compared methylation levels at IGF2, GNAS, INSIGF, and LEP between preterm infants <32 weeks small for gestational age (SGA) and those appropriate for gestational age (AGA) and did not find significant alterations of the methylation status at these loci. Another study on SGA pregnancies reported on 11p15 methylation abnormalities detected in placental tissue of SGA compared to AGA pregnancies, whereas no such differences were seen in DNA from corresponding neonatal blood samples [45].

Somewhat unexpectedly, the observed intra-individual correlations of CpG methylation levels within single 11p15 gene regions (Spearman’s ρ maximum 0.814 (saliva)/0.748 (blood)) were only modest, which may be partially explained by the relatively small number of included CpG sites per region (n = 2). We are aware that methods other than the SNuPE IP RP HPLC assay used in our study may have been advantageous in terms of the quantity of CpG sites to be analyzed. However, considering presumed (and observed) effects of only a few percent variation of locus-specific methylation levels, we regarded this highly quantitative method [21-23] as the method of choice.

Similar to findings from other recent studies analyzing larger amounts of CpG sites at the 11p15 region [40-43], intra-individual correlations between CpG sites across different 11p15 dmr*s* were, if detectable, only weak (Spearman’s ρ maximum 0.335). Together with significant intra-twin pair correlations observed in our cohort and previous studies this may indicate that locus-specific methylation levels are regulated by their local genetic background [15,40-43]. On the other hand, comparing intra-twin pair differences at a specific region against the differences arising at other regions revealed a small number of significant correlations, almost all of which, notably, showed positive correlation coefficients (see Figure 5). Thus, methylation differences within and between regions in our twin cohort appear to arise with a consistent directionality, indicating that environmental factors may affect the 11p15 epigenome in a more global way.

We noted substantial intra-individual differences between methylation measurements from either saliva- or blood-derived DNA. Variance of locus-specific methylation as well as intra-twin pair differences were generally higher in saliva DNA, and only two out of eight CpG sites (LINE-1 CpG SN13, H19 CpG SN5) showed significant inter-tissue correlations between blood and saliva samples. The issue of epigenotypical variation across different tissue types has been discussed intensively during recent years. Although inter-tissue correlations of region-specific methylation as well as robust interactions between epigenotype and genetic background have been reported for several non-imprinted and imprinted regions including 11p15 [15,40-43,46,47], systematic approaches analyzing larger numbers of tissues and loci strongly endorse the concept that methylation patterns at a variety of regions are commonly influenced by tissue-specific and environmental factors [41,46-50]. Furthermore, DNA samples derived from oral mucosa epithelium may be particularly susceptible to short-term changes and environmental effects [51,52]. We are aware that biological variation resulting from differing cell type composition in saliva samples (mucosa cells and leukocytes) and other biotechnical artifacts related to the saliva sampling method cannot be fully excluded. In a previous project on the same 20 twin pairs, we repeated all experimental steps including DNA preparation, bisulfite treatment, PCR reactions and site-specific SNuPE IP RP HPLC for all 40 saliva samples, showing intra-individual variation of below 5% [15]. Finally, the fact that intra-twin pair methylation differences in blood and saliva DNA appear to arise with a consistent directionality (see Figure 5, right panel) may be indicative of variation due to physiological changes rather than technical artifacts.

We did not find significant relations between prenatal growth discordance and IGF-II serum levels. Generally, IGF-II is known as a potent promoter of prenatal growth as demonstrated in animal models and naturally occurring 11p15 imprinting disorders in humans [6,53]. Within healthy populations, circulating IGF-II levels as well as common IGF2 gene polymorphisms have been associated with size at birth [54,55]. However, little is known about the developmental plasticity of IGF-II and there are only a few studies on IGF-II serum levels in growth-discordant monozygotic twin pairs so far. In a cohort of 13 TTTS twin pairs, Bajoria and colleagues [56] found significantly lower IGF-II concentrations in cord blood samples of TTTS donors as compared to both recipients and a control group of monochorionic twin pairs without TTTS. In contrast, IGF-II serum levels in our twin cohort were comparable between donors and recipients both at birth [14] and at age 4, whereas serum levels of *IGF-I* were strongly related to intrauterine growth and subsequent catch-up growth [19]. This is in line with most studies in SGA infants associating prenatal growth restriction with decreased *IGF-I* levels [57,58], although some impact also on IGF-II has been discussed [59,60]. In our cohort there was also no relationship between methylation at any of the analyzed CpG sites at 11p15 and circulating IGF-II concentrations. However, normal serum IGF-II levels are seen even in patients with SRS due to 11p15 imprinting defects, which may reflect the non-imprinted biallelic postnatal IGF2 expression in the liver [61-63].

## Conclusion

In summary, we have analyzed locus-specific methylation levels at different 11p15 regions in a cohort of 20 monozygotic twin pairs with discordant intrauterine development due to severe TTTS. Slight but significant methylation differences within the twin pairs were observed at only one (IGF2 dmr0) out of three analyzed 11p15 regions. Although a certain susceptibility of the postnatal IGF2 dmr0 methylation pattern to environmental factors during early developmental stages was also reported by other groups [9,10], it is not known whether such small methylation changes (IGF2 dmr0 SN3 mean difference in our cohort: + 1.87% in donors) can significantly alter the complex regulation of gene transcription at 11p15. We conclude that severe alteration in prenatal blood supply due to TTTS appears to leave only weak, if any, locus-specific epigenetic marks at the analyzed 11p15 regions.

## Abbreviations

IGF: insulin-like growth factor; IP RP HPLC: ion pair reverse-phase high performance liquid chromatography; PCR: polymerase chain reaction; SDS: standard deviation score; SGA: small for gestational age; SNuPE: single nucleotide primer extension reaction; SRS: Silver-Russell syndrome; TTTS: twin-to-twin transfusion syndrome.

## Competing interests

The authors declare that they have no competing interests.

## Authors’ contributions

FS, BG, PB, KH, JO, OEM and JW designed the study. KH performed the fetoscopic laser therapy. FS, BG, SS and KH collected patient data and samples. FS and OEM performed the experiments. FS, BG, OEM, and JW analyzed the data. FS wrote the paper. All authors read and approved the final manuscript.

## Supplementary Material

Additional file 1: Table S1 Primer sequences and exact CpG position. **Table S2.** Auxological parameters and intra- twin pair methylation differences according to the concordance/discordance status at birth. **Table S3.** Inter-twin correlations of locus-specific methylation levels according to the concordance/discordance status at birth. Click here for file

Additional file 2: Figure S1 Inter-twin correlation of insulin-like growth factor (IGF)-II serum levels at age 4. IGF-II serum levels at age 4 showing significant inter-twin correlations (total cohort Pearson R = 0.79, *P* < 0.01). Note that IGF-II serum levels in pairs discordant for birth weight and/or length at birth seem to correlate even stronger (filled circles/solid line = concordant pairs, R = 0.77, *P* = 0.016; open circles/dotted line = discordant pairs, R = 0.89, *P* < 0.01), although the intra-twin pair variation among the two groups did not differ significantly (*P* > 0.2). Click here for file
