# Supplementary material for: 11p15 DNA-methylation analysis in monozygotic twins with discordant intrauterine development due to severe twin-to-twin transfusion syndrome
Source: Clin Epigenetics. 2014 Mar 28;6(1):6. doi: 10.1186/1868-7083-6-6 (PMC3986638; doi:10.1186/1868-7083-6-6)
Supplement: Additional file 1: Table S1 — Primer sequences and exact CpG position. Table S2. Auxological parameters and intra- twin pair methylation differences according to the concordance/discordance status at birth. Table S3. Inter-twin correlations of locus-specific methylation levels according to the concordance/discordance status at birth. [file 1868-7083-6-6-S1.doc]

Additional file 1: Table S1

| **Region** | **Amplification primer (5’3’)** | **SNuPE primer (5’3’)** | **CpG** | **Position (accession #)** |
| --- | --- | --- | --- | --- |
| **IGF2 dmr0**  *IGF2dmr0bis_1 x*  *IGF2dmr0bis_rR*  *IGF2dmr0bis_F x*  *IGFdmr0bis_2* | *outer PCR pair*  GAGGTATTTTGGGGTTAAGGT  ACCTCCTCCACCTCCAAACAC  *nested PCR pair*  GGGAATGTTTATTTATGTATGAAGTT  CCCAACAAAAACCACTAAACAC | TTTATTTTTTTAGGAAGTATAGTTA  TTTTTTATTGGTTT | SN1  SN3 | 747 (Y13633)  766 |
| **KCNQ1OT1 ***  *KCNQ1bis_1 x*  *KCNQ1bis_4*  *KCNQ1bis_2 x*  *KCNQ1bis_3* | *outer PCR pair*  CCTCACACCCAACCAATACC  GTTTTGGTAGGATTTTGTTGAGGAG  *nested PCR pair*  CAAACCTTCCCCTACTACC  GTTGAGGAGTTTTTTGGAGGT | TTTTTTGGAGGTT  TTTTTTATTTGTTGATTGGG | SN16  SN1 | 67521 (U90095)  67417 |
| **H19 prom**  *H19bis_F1 x*  *H19bis_R1* | TTTTTGGTAGGTATAGAGTT  AAACCATAACACTAAAACCC | TGGTTGTAGTTGTGGAAT  TTTTTTTTTTAATTGGGGTT | SN5  SN12 | 6180 (AF087017)  6247 |
| **LINE-1**  *Bis_L1_F1 x*  *Bis_L1_R2* | ATTTTATTAGGGAGTGTTAGATAGTG  TTAAACTATAATAAACTCCACCCAAT | GGGAGTGTTAGATAGTGGG  TTTGGGTTTTTTTTATT | SN1  SN13 | 136 (X52235)  283 |

* Note that methylation of CpG sites SN16 and SN1 at the KCNQ1OT1 region was analyzed using the corresponding 3’5’ bisulfite DNA strand, since no suitable primer combinations were found on the 5’3’ bisulfite DNA strand. All oligonucleotide sequences (including KCNQ1OT1) are displayed in 5’3’ direction.

***Additional file 1: Table S2***

|  | **weight /length**  ***concordant* at birth**  (n = 9 pairs) | **weight/length**  ***discordant* at birth**  (n = 11 pairs) | ***p*** |
| --- | --- | --- | --- |
| gestational age at laser treatment [wks] | 21.13 ± 2.42 | 20.82 ± 2.21 | 0.715 |
| gestational age at birth [wks] | 34.47 ± 2.07 | 34.60 ± 2.28 | 0.847 |
| Diff. birth weight [g] | 90.56 ± 90.91 | 582.36 ± 247.32 | **<0.001** |
| Diff. birth weight SDS | 0.21 ± 0.21 | 1.44 ± 0.70 | **<0.001** |
| Diff. birth length SDS | 0.51 ± 0.54 | 1.29 ± 1.00 | **0.041** |
| Diff. IGF-II (cord blood) | 30.13 ± 69.52 | -30.38 ± 49.72 | 0.067 |
| Diff. IGF-II (at age 4 yrs) | 2.56 ± 85.70 | -14.55 ± 38.03 | 0.590 |
| ***Intra-twinpair methylation difference [%] saliva*** | | | |
| IGF2 dmr0 SN1 | -0.35 ± 10.15 | 2.28 ± 8.48 | 0.595 |
| IGF2 dmr0 SN3 | -4.46 ± 12.98 | -0.54 ± 5.31 | 0.373 |
| KCNQ1OT1 SN16 | -3.61 ± 15.00 | 0.64 ± 16.54 | 0.590 |
| KCNQ1OT1 SN1 | -3.00 ± 21.55 | 4.05 ± 17.33 | 0.480 |
| H19 prom SN5 | -0.59 ± 1.66 | 2.79 ± 9.29 | 0.446 |
| H19 prom SN12 | 4.23 ± 10.34 | 4.19 ± 13.14 | 0.996 |
| LINE-1 SN1 | 0.09 ± 1.78 | 0.30 ± 0.81 | 0.748 |
| LINE-1 SN13 | -0.77 ± 1.01 | 0.12 ± 0.98 | 0.083 |
| ***Intra-twinpair methylation difference [%] blood*** | | | |
| IGF2 dmr0 SN1 | -0.89 ± 1.68 | 0.12 ± 1.59 | 0.185 |
| IGF2 dmr0 SN3 | -2.05 ± 2.96 | -1.71 ± 3.27 | 0.814 |
| KCNQ1OT1 SN16 | -0.49 ± 2.22 | -0.47 ± 2.11 | 0.980 |
| KCNQ1OT1 SN1 | -0.60 ± 2.51 | -0.41 ± 1.33 | 0.827 |
| H19 prom SN5 | -0.65 ± 3.76 | 0.88 ± 2.03 | 0.263 |
| H19 prom SN12 | -0.37 ± 4.20 | -0.10 ± 1.61 | 0.842 |
| LINE-1 SN1 | -0.07 ± 0.58 | -0.25 ± 0.84 | 0.596 |
| LINE-1 SN13 | -0.02 ± 0.77 | 0.02 ± 0.85 | 0.931 |

**Comparison of auxological parameters and intra-twinpair methylation differences according to the concordance / discordance status at birth.**  Note that variation of methylation was generally higher in saliva compared to blood –derived DNA . Values are given as mean ± SD; Difference values represent the arithmetic mean (± SD) of intra-pair differences (recipient/*minuend* – donator/*subtrahend*).

***Additional file 1: Table S3***

(A) Inter-twin correlations / Saliva

|  | **IGF2 dmr0** | | **KCNQ1OT1** | | **H19 prom** | | **LINE-1 repeats** | |
| --- | --- | --- | --- | --- | --- | --- | --- | --- |
|  | **SN1** | **SN3** | **SN16** | **SN1** | **SN5** | **SN12** | **SN1** | **SN13** |
| **All**  **(n=16 pairs)** | **0.539*** | 0.217 | 0.235 | -0.017 | -0.118 | -0.366 | 0.039 | **0.676**** |
| **BW/BL-concordant**  **pairs (n=8)** | -0.372 | 0.141 | 0.407 | -0.254 | 0.482 | -0.565 | -0.306 | **0.747*** |
| **BW/BL-discordant**  **Pairs (n=8)** | **0.884**** | **0.652^§^** | 0.072 | 0.187 | -0.210 | -0.428 | 0.517 | **0.687*** |

Spearman’s rank correlation coefficients; *p<0.05; **p<0.01; ^§^p=0.057

(B) Inter-twin correlations / Blood

|  | **IGF2 dmr0** | | **KCNQ1OT1** | | **H19 prom** | | **LINE-1 repeats** | |
| --- | --- | --- | --- | --- | --- | --- | --- | --- |
|  | **SN1** | **SN3** | **SN16** | **SN1** | **SN5** | **SN12** | **SN1** | **SN13** |
| **All**  **(n=20 pairs)** | **0.626**** | **0.578**** | 0.006 | 0.144 | **0.892**** | **0.965**** | **0.436^$^** | **0.474*** |
| **BW/BL-concordant**  **pairs (n=9)** | **0.716*** | 0.586 | -0.389 | -0.622 | **0.909**** | **0.923**** | **0.773*** | 0.624 |
| **BW/BL-discordant**  **pairs (n=11)** | **0.610*** | 0.566 | 0.211 | **0.667*** | **0.862**** | **0.991**** | -0.313 | 0.437 |

Spearman’s rank correlation coefficients; *p<0.05; **p<0.01; ^$^p=0.055
