# Supplementary figures and images for: 11p15 DNA-methylation analysis in monozygotic twins with discordant intrauterine development due to severe twin-to-twin transfusion syndrome
Source: Clin Epigenetics. 2014 Mar 28;6(1):6. doi: 10.1186/1868-7083-6-6 (PMC3986638; doi:10.1186/1868-7083-6-6)

## Slide 1
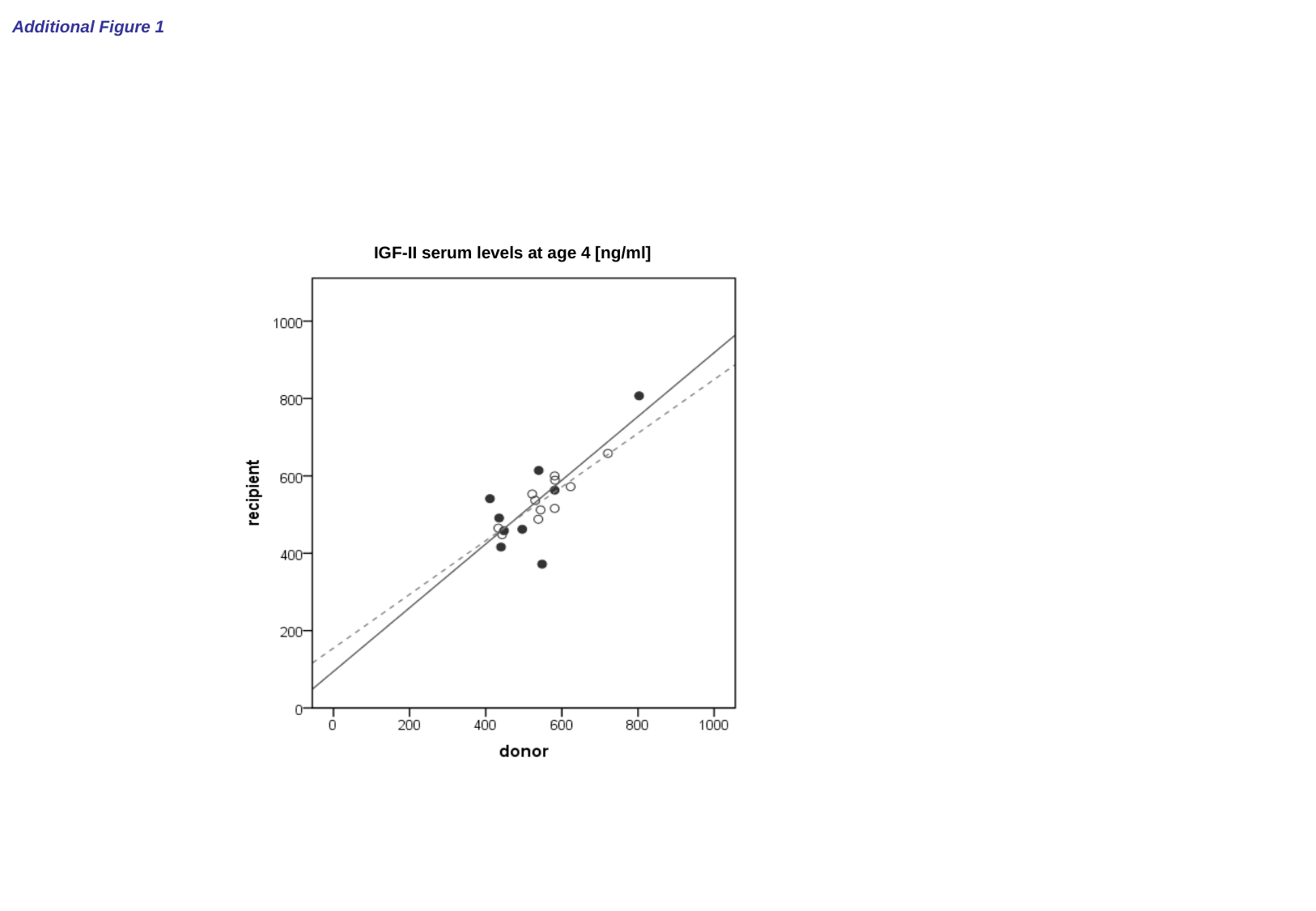

Additional Figure 1
IGF-II serum levels at age 4 [ng/ml]

Supplement: Additional file 2: Figure S1 — Inter-twin correlation of insulin-like growth factor (IGF)-II serum levels at age 4. IGF-II serum levels at age 4 showing significant inter-twin correlations (total cohort Pearson R = 0.79, P < 0.01). Note that IGF-II serum levels in pairs discordant for birth weight and/or length at birth seem to correlate even stronger (filled circles/solid line = concordant pairs, R = 0.77, P = 0.016; open circles/dotted line = discordant pairs, R = 0.89, P < 0.01), although the intra-twin pair variation among the two groups did not differ significantly (P > 0.2). [file 1868-7083-6-6-S2.ppt]
